# Evaluation of Banana Leaves (Musa paradisiaca) as an Alternative Wound Dressing Material Compared to Conventional Petroleum Jelly Gauze Dressing in Contused, Lacerated and Sutured Wounds Over the Head, Neck and Face Region

**DOI:** 10.7759/cureus.18552

**Published:** 2021-10-06

**Authors:** Swapnil Chendake, Tejraj Kale, Yash Manavadaria, Abhishek S Motimath

**Affiliations:** 1 Department of Oral & Maxillofacial Surgery, Dr. D.Y. Patil Medical College and Hospital, Kolhapur, IND; 2 Department of Oral & Maxillofacial Surgery, KLE VK Institute of Dental Sciences, Belagavi, IND

**Keywords:** wound, petroleum jelly, banana leaves, dressings, head and neck trauma

## Abstract

Aim

To evaluate the effectiveness of banana leaf dressing in patients with contused, lacerated and sutured wounds over the head, neck and face region with respect to pain during dressing change, patient comfort, status of wound bed during dressing change and handling characteristics in comparison with petroleum jelly gauze dressing.

Methods and materials

Sixty patients were included, out of which 30 patients were used as controls with petroleum jelly gauze dressings and 30 patients as study participants with banana leaf dressings. Pain on dressing change, handling characteristics of dressing material, patient comfort and status of wound bed on every dressing change were assessed.

Results

Properties of both banana leaves and petroleum jelly gauze dressings were parallel in all aspects, except pain on dressing change which was less with banana leaf dressings and had statistically significant value (p>0.001).

Conclusions

Banana leaves (*Musa paradisiaca*) can become an alternative choice of wound dressing material in contused, lacerated and sutured wounds over the head, neck and face region as they proved to cause less pain and trauma during dressing change and have other advantages such as cost and availability, comfort and ease of handling the dressing by health professionals.

## Introduction

The face, being the most exposed part of the body, is particularly vulnerable to traumatic injuries. In rural areas, road traffic accidents are a major cause of maxillofacial trauma. Secondary to facial fractures there are associated soft tissue injuries which need to be taken care of during hospital stay.

Facial soft tissue injuries are of different types which include contusions, lacerations, abrasions, avulsions and bite injuries. After primary management of soft tissue injuries, the wound needs to be protected from the external environment for its optimal healing.

Wound dressings are local therapeutic agents used to create an optimal environment for healing, with specific properties according to the type and physiologic healing stage of the wounds [1].

There is an increasing consensus that pain is always an important factor for patients suffering from many different wound types [2]. Dressing change is the time when the patient experiences most of the pain.

Dressings are categorized depending upon their physical composition and mechanism of action. Categories of wound dressings are knitted viscose and gauze, tulle (gauze impregnated with petroleum jelly), semi-permeable film, hydro-colloid, hydro-gels, alginate, bead and foam dressings [3].

Gauze dressing when used over the wound does not create a moist environment for wound healing. It can delay or interrupt the wound healing process by drying and adhering to the wound bed, and thus causing damage to the tissue when it is removed. The remaining dressing types previously listed have less risk of adhering to the wound and are referred to as non-adherent or low-adherent dressing materials [2,3].

The ideal wound dressing material is one that can maintain a moist environment at the wound interface and act as a barrier to microorganisms, while also being non-adherent, non-toxic, non-allergenic, non-sensitizing, and easily removed without trauma and discomfort [4].

Dressing materials available today possess most of these properties, but they are expensive, especially when required for longer duration which adds more financial burden to the patient, so their use in developing countries where all the patients cannot afford regular dressing changes with expensive dressings is limited [4].

Banana leaves have been used conventionally as wound dressing material in India as part of an ancient medical practice. With regard to its properties, like a large surface area and a waxy and cool surface, they were used in treatment of smallpox. Recent studies are done for scientific assessment of banana leaves as a wound dressing material in research and clinical settings [1,4].

Banana leaves can become an alternative choice of dressing material in hospital and clinical settings where cost and availability of dressing material are important issues [5,6].

To the best of our knowledge there is no literature on the use of banana leaf dressing in the head and neck region. Based on the good properties proved in studies on other areas, this is the first study where banana leaf was used as an alternative dressing material in the head and neck region to treat wounds.

The present study was performed to evaluate the efficacy of autoclaved banana leaves (*Musa paradisiaca*) as an effective alternative dressing material in contused, lacerated or sutured wounds over the head, neck and face in trauma victims and compare them with conventional petroleum jelly gauze (tulle) dressing.

## Materials and methods

Study population

The study population consisted of 60 patients who reported to KLE'S Dr. Prabhakar Kore Hospital and Research Centre, Belagavi, Karnataka, India.

Materials

Materials required for the procedure included banana leaves from the species *M. paradisiaca* and a small gauze and tissue plaster to hold the dressing in position.

Inclusion criteria

Patients in the age group between 10-50 years of either sex with contused, lacerated, sutured wounds of size 3-8 cm in length and 0.5-1.5 cm in depth over the head, neck and face region admitted in KLE’S Dr. Prabhakar Kore Hospital and Research Centre, Belagavi, in the period of September 2014 to May 2016 were included in the study after procuring approval from the Research and Ethical Committee, KLE VK Institute of Dental Sciences, Belagavi, with approval number 797.

Exclusion criteria

Immunocompromised patients, psychiatric patients, substance abusers (including alcohol and cigarettes) and those with history of/undergoing chemotherapy or radiotherapy were excluded from the study. Moreover, wounds showing signs of maceration were also excluded. 

Data collection

A simple random sampling technique was used for data collection. Written informed consent was taken from the patient and a standard proforma was used to collect the necessary information regarding each case.

Statistical analysis

The data collected were analyzed using chi-square test and stratified according to age, sex, pain score, status of wound, handling characteristics and comfort characteristics.

Procedure

**Figure 1:**
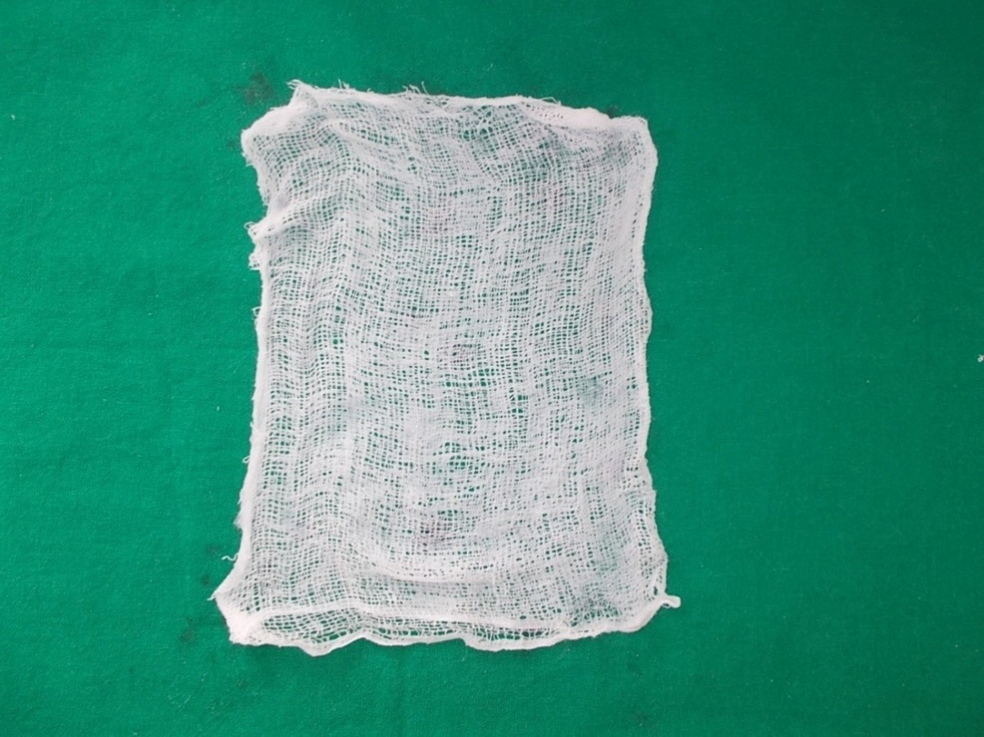
Petroleum jelly gauze dressing.

**Figure 2:**
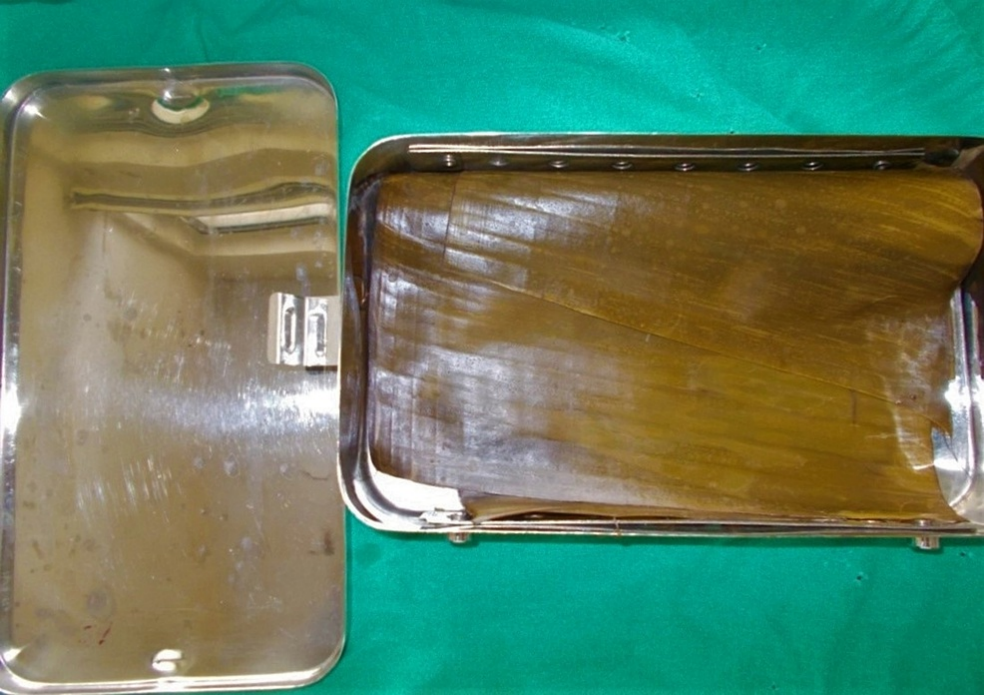
Autoclaved banana leaf.

Sixty patients, out of which 30 patients were controls in which petroleum jelly gauze dressing (Figure 1) was used and 30 patients were the study sample in which banana leaf dressing (Figure 2) was used.

Freshly cut banana leaves from the species *M. paradisiaca* obtained from an agriculture farm were washed with detergent or soap to remove all the dust. These banana leaves were autoclaved at 15 lbs/in^2^ pressure for 121°C for 15 minutes [6,7]. A small gauze and tissue plaster was used to hold the dressing in position.

Pain during dressing change was assessed using the NIH pain scale. The wound bed was evaluated by using the Exudate Interaction Scale. Handling characteristics for each dressing material were noted as Easy to Handle: Score 1, Difficult To Handle: Score 2. Patient comfort characteristics were noted as Comfortable Feeling: Score 1, Minor Discomfort: Score 2, Extreme Discomfort: Score 3.

The dressing was changed daily for seven days and patients were recalled for final follow up on the 14^th^ day.

## Results

Age distribution

Table 1 shows age wise distribution of the study subjects. A total of 60 patients were included in the study. Thirty were given banana leaf dressing and 30 petroleum jelly gauze as dressing.

**Table 1:** Age wise distribution of study subjects.

Type of dressing	n	Mean Age	Std. Deviation (+)
Banana Leaf	30	29.03	10.397
Petroleum jelly Gauze	30	33.67	11.127
Unpaired t-test p value = 0.10 (Not significant)			

Sex distribution

Table 2 shows distribution of study subjects according to gender.

**Table 2:** Distribution of the study subjects according to gender.

Type of dressing	Gender		Frequency	Percentage (%)
Banana Leaf		Male	25	83.3
		Female	5	16.7
		Total	30	100.0
Petroleum jelly Gauze		Male	22	73.3
		Female	8	26.7
		Total	30	100.0
Chi-Square Value = 0.88 p value = 0.347 (Not significant)				

Association of type of dressing and pain score

Table 3 shows distribution of study subjects according to the pain score. Patients with banana leaf dressings experienced mild to no pain whereas patients with petroleum jelly gauze dressings experienced mild pain.

**Table 3:** Distribution of the study subjects according to pain score.

Type of dressing	Pain score		Frequency	Percentage (%)
Banana Leaf		No pain	24	80.0
		Mild Pain	6	20.0
		Moderate pain	0	0.0
		Severe pain	0	0.0
		Total	30	100.0
Petroleum jelly Gauze		No pain	0	0.0
		Mild Pain	30	100.0
		Moderate pain	0	0.0
		Severe pain	0	0.0
		Total	30	100.0

Association of type of dressing and status of wound bed

Table 4 shows distribution of study subjects according to status of the wound bed. For both the dressings wound bed was observed to be moist during every dressing change.

**Table 4:** Distribution of the study subjects according to status of the wound.

Type of dressing	Status of wound		Frequency	Percentage (%)
Banana Leaf		Dry	0	0.0
		Moist	30	100.0
		Wet	0	0.0
		Saturated	0	0.0
		Leaking	0	0.0
		Total	30	100.0
Petroleum jelly Gauze		Dry	0	0.0
		Moist	30	100.0
		Wet	0	0.0
		Saturated	0	0.0
		Leaking	0	0.0
		Total	30	100.0

Association of type of dressing and handling characteristics

Table 5 shows distribution of study subjects according to handling characteristics. Both dressings were easy to handle.

**Table 5:** Distribution of the study subjects according to handling characteristics.

Type of dressing	Handling Characteristics		Frequency	Percentage (%)
Banana Leaf		Easy to Handle	30	100.0
		Difficult to Handle	0	0.0
		Total	30	100.0
Petroleum jelly Gauze		Easy to Handle	30	100.0
		Difficult to Handle	0	0.0
		Total	30	100.0

Association of type of dressing and comfort characteristics

Table 6 shows distribution of study subjects according to comfort characteristics. Both dressing materials were comfortable to the patients.

**Table 6:** Distribution of the study participants according to comfort characteristics.

Type of dressing	Comfort Characteristics		Frequency	Percentage (%)
Banana Leaf		Comfortable Feeling	30	100.0
		Minor Discomfort	0	0.0
		Extreme Discomfort	0	0.0
		Total	30	100.0
Petroleum jelly Gauze		Comfortable Feeling	30	100.0
		Minor Discomfort	0	0.0
		Extreme Discomfort	0	0.0
		Total	30	100.0

Analysis of type of dressing and pain score

Table 7 shows analysis of values of pain score which shows that banana leaf dressing causes comparatively less pain than petroleum jelly gauze dressing (p<0.001).

**Table 7:** Association between the type of dressing and pain score. *p<0.05

Type of dressing	Pain score	Frequency	Percentage (%)	Chi-Square value	p value
Banana Leaf	No pain	24	80.0	40	<0.001*
	Mild Pain	6	20.0		
	Moderate pain	0	0.0		
	Severe pain	0	0.0		
	Total	30	100.0		
Petroleum jelly Gauze	No pain	0	0		
	Mild Pain	30	100.0		
	Moderate pain	0	0		
	Severe pain	0	0		
	Total	30	100.0		

## Discussion

Road traffic accident is the major cause of the facial trauma. Injuries to the head and face occur in 70% of all automobile accidents [8]. In India, due to high-speed automobiles and poor road conditions, incidence of road traffic accidents and injuries to the maxillofacial skeleton are increasing alarmingly.

Facial contused and lacerated wounds require primary management and regular dressing change to protect the wound from microbial contamination and to facilitate healing.

Pain is always an important issue during dressing change. Occlusive conventional dressings tend to dry and adhere to the wound bed so, during removal it causes secondary trauma to the wound and ultimately wound healing is delayed [2].

The purpose of this randomized controlled trial was to establish the property of banana leaf (*M. paradisiaca*) as an alternative wound dressing material to conventional petroleum jelly gauze dressing in contused, lacerated and sutured wounds over the head, neck and face region.

In our study, petroleum jelly gauze dressing was found to cause tissue trauma when removed from the wound bed during dressing change. Trauma and pain during dressing change were still minimal as petroleum jelly itself is very low adherent. Banana leaf dressing on the other hand is completely non-adherent; pain and trauma during dressing change were very lower then petroleum jelly gauze dressing and had statistically significant value (p<0.001). A study was conducted by Moffatt et al. in 2003 for understanding wound pain and trauma during dressing change. They suggested that dressing removal is considered to be the time of most pain, dried out dressings and adherent products are most likely to cause pain and trauma during dressing change, so the dressing should be non-traumatic and should prevent tissue trauma [2]. So as per our study, banana leaf dressing proved out to be less painful during dressing removal, causing minimal damage to the newly formed epidermis during dressing removal. Patients were more comfortable during every dressing change than with conventional petroleum jelly gauze dressing.

We have a plethora of modern dressing materials which possess all the properties of ideal dressing material, but in developing countries like India, cost and availability of dressing materials are of major concern [1].

Gore and Akolekar in 2003 used autoclaved banana leaves over partial thickness burns and skin graft donor areas. They observed that banana leaf dressing results in early epithelization and minimal pain during dressing change. The results of their study helped to establish banana leaves as an effective alternative dressing material with regards to its unlimited source and favorable properties [5,6]. Our results are parallel to the study by Gore and Akolekar.

Guenova et al. in 2013 used banana leaf dressing in post-surgical wounds. Patients who underwent surgeries for webbed fingers, phimosis, penile candidiasis hernia, fibroadenoma, etc. were candidates selected for banana leaf dressing. Banana leaf dressings were prepared by autoclaving and used post-surgically to cover the wound. Follow-ups were done after 24 hours, seven days and 14 days. The authors suggested that banana leaf dressings are beneficial and well tolerated by the patient [4]. 

We used autoclaving as the technique for the sterilization of banana leaves. Banana leaves were autoclaved at a pressure of 15 lbs/in^2^ at 121°C for 15 minutes. Autoclaving assured us complete sterilization. There were no signs of infection, tissue dehiscence or suture breakdown in all our study subjects.

Srinivas et al. compared various techniques of sterilization of banana leaves. He found that autoclaving is the only effective method for sterilization [7]. Thus, we can say that autoclaving does not affect the properties of banana leaves as dressing material and also assures us complete sterilization.

Moreover, Gore, Umakumar, and Iyer compared polyethelene surgical drape and banana leaf dressing for split thickness skin graft (STSG) donor area. They observed that both dressing materials are equally effective except polyethelene surgical drapes caused less pain on dressing removal than banana leaves [9]. On the other hand, we observed in our study that banana leaf dressing is superior to petroleum jelly gauze dressing. Use of polyethelene surgical drape over the facial contused, lacerated wounds and its comparison with banana leaf dressing requires further evaluation in clinical settings.

Banana leaf dressing can become an alternative choice of dressing material in developing countries like India where cost and availability of dressing material are important issues. Banana leaves are easily available, grown throughout the year, possess all the properties of ideal wound dressing material, and are easy to prepare and use. Therefore, we strongly recommend use of banana leaf dressings over facial wounds.

Our observations during the study

The shelf life of autoclaved banana leaves is around seven to 10 days, after that, fungal growth is seen over the leaf. Accordingly, dressing should be prepared and used within three to four days and later discarded. Dressing needs to be prepared fresh before use and should be stored in aseptic conditions. The midrib portion of the leaf is cut and autoclaved. Awareness amongst patients is necessary regarding use of banana leaf dressing. Banana leaf dressing being completely non-adherent tends to slip. Thus, it requires proper support with gauze and sticking plaster. Banana leaves should be washed thoroughly to remove dust particles, dirt and pesticides and then autoclaved.

## Conclusions

Statistical analysis of the study data revealed that properties of both banana leaves and petroleum jelly gauze dressings were parallel in all aspects, except pain on dressing change which was less with banana leaf dressings than petroleum jelly gauze dressings and had statistically significant value (p>0.001).

To conclude, banana leaves (*M. paradisiaca*) can become an alternative choice of wound dressing material in contused, lacerated and sutured wounds over the head, neck and face region. Banana leaf dressing was observed to be more effective with respect to pain and trauma during dressing change, cost and availability, comfort, ease of handling the dressing by health professionals and thus regular use of banana leaf dressing can be emphasized. Since banana leaf dressing is parallel/superior to Vaseline gauze dressing, we recommend usage of banana leaf on a regular basis in all surgical units, due to its excellent dressing properties.

Since the study is solely dependent on trauma patients reporting to hospital casualty, the number of patients included in this short-term study is low. Moreover, patients with abrasions and tissue loss were also excluded from the study to eliminate sampling bias. Taking this into consideration, a bigger prospective study will be conducted later for an extended period of time.
